# Hypoxia and estrogen are functionally equivalent in breast cancer-endothelial cell interdependence

**DOI:** 10.1186/1476-4598-11-80

**Published:** 2012-10-22

**Authors:** Andrea L George, Shilpi Rajoria, Robert Suriano, Abraham Mittleman, Raj K Tiwari

**Affiliations:** 1 Department of Microbiology and Immunology, New York Medical College, Valhalla, NY, 10595, USA

**Keywords:** Estrogen, Hypoxia, Neovasculogenesis, Vascular endothelial growth factor, Hypoxia inducible factor, Breast cancer

## Abstract

**Background:**

Rapid breast tumor development relies on formation of new vasculature to supply the growing malignancy with oxygenated blood. Previously we found that estrogen aided in this neovasculogenesis via recruitment of bone marrow derived endothelial progenitor cells (BM-EPCs), leading to increased vessel formation and vascular endothelial growth factor (VEGF) production *in vivo*. However, the cellular mechanism of this induction and the signaling pathways involved need elucidation.

**Results:**

Using the murine mammary cell line TG1-1 we observed estrogen (E_2_) lead to an up regulation of hypoxia inducible factor-1 (HIF-1), an effect abrogated by the anti-estrogen Fulvestrant and the HIF-1 inhibitor YC-1 (3-(5’-hydroxymethyl-2’-furyl)-1-benzylindazole) suggesting the interchangeability of hypoxia and estrogen mediated effects. Estrogen modulation of HIF-1 and subsequent effects on endothelial cells is dependent on the Akt/PI3K pathway and protein synthesis as validated by the use of the inhibitors wortmannin and cycloheximide which abrogated estrogen’s effects respectively. Estrogen treated TG1-1 cells secreted higher levels of VEGF which were comparable to secreted levels from cells grown under hypoxic conditions. Soluble factors in conditioned media from E_2_ treated breast cancer cells also lead to migration and tube formation of human umbilical vein endothelial cells (HUVEC) *in vitro*.

**Conclusions:**

Our data provide evidence that estrogen signaling mediates the tumor vasculogenic process required for breast cancer progression and involves a key regulator of the hypoxia signaling pathway. Further, hypoxia and estrogen are interchangeable as both similarly modulate epithelial-endothelial cell interaction.

## Background

Breast cancer is recognized as the most common type of cancer in women and its development is associated with many risk factors such as diet, alcohol consumption, child bearing, breast feeding, oral contraception, as well as underlying genetic predisposition. Epidemiological studies show a rapid increase in breast cancer incidence during reproductive years that tapers around age 50, corresponding to the onset of menopause, and studies of postmenopausal breast cancer patients have found a higher level of estrogen in breast tissue compared to healthy patient tissue [1-6]. Taken together with the fact that 60-70% of human breast cancers are estrogen receptor-alpha positive [7], the evidence suggests an etiological significance of estrogen in breast cancer initiation and progression.

Estrogen is a sex steroid hormone produced mostly by the ovaries in women; however other tissues, including adipose, are also able to synthesize estrogen. There are a total of nine estrogens in humans of which 17β-Estradiol (E_2_) is the most abundant in circulation and the most biologically active [8]. Estrogen mediates its effects by binding to its cognate estrogen receptor(s), either estrogen receptor alpha (ERα) or estrogen receptor beta (ERβ), leading to ER dimerization and association with various co-factors. Once formed, the complex translocates to the nucleus where it acts as a transcription factor by binding to the estrogen response elements (EREs) at the promoters of estrogen responsive genes [9,10]. Besides this classical pathway, estrogen can also regulate gene transcription in ERE independent as well as nongenomic pathways by binding to membrane associated estrogen receptor leading to signaling via the PI3K/AKT pathway [11,12]. In addition to its normal physiological roles, estrogen is also implicated in breast cancer initiation and progression. Estrogen-ER interactions have been observed to increase cell survival by signaling through the AKT pathway which leads to suppression of TNF-α induced apoptosis [13]. Estrogen-ER signaling also induces cell proliferation by activation of the PI3K pathway, observed in breast cancer cell lines including ER^+^ MCF-7 cells, but not the ER^-^ MDA-MB-231 cell line [14]. Interestingly, estrogen is also capable of contributing to breast cancer progression by a novel role, via modulation of proteins involved in hypoxia signaling, namely hypoxia inducible factor 1 (HIF-1).

HIF-1 is also a heterodimeric transcription factor, consisting of the oxygen dependent alpha subunit and the constitutively expressed beta subunit. During normoxia, HIF-1α is rapidly degraded via the proteasomal pathway, however during hypoxia, HIF-1α is stabilized and binds HIF-1β (aryl hydrocarbon receptor nuclear translocator, ARNT), forming a transcriptional complex which translocates to the nucleus where, with other protein co-factors, it binds hypoxia responsive elements (HRE) [15]. Binding of HIF-1 to target genes leads to transcription of proangiogenic proteins including erythropoietin and VEGF, which are essential for formation of new blood vessels, or neovasculogenesis [16]. Further, the chemotactic protein stromal derived factor 1 (SDF-1) is also hypoxia responsive, leading to development of a chemotactic gradient for bone marrow derived cells that express the cognate receptor CXCR4 [17-19]. In a rat uterine model, estrogen was observed to increase HIF-1α levels *in vivo* and this induction lead to an increase in VEGF expression that was abrogated by PI3K inhibitors but not MAPK inhibitors [20,21]. Chromatin immunoprecipitation assays found that this estrogen treatment lead to binding of both ER and HIF-1 to VEGF promoters [22]. E_2_ also lead to up regulation of HIF-1 in ovarian cancer cells in a PI3K dependent manner [23,24]. ER positive breast cancers have also been linked to an increased expression of HIF-1 and correlated with a more metastatic phenotype [25,26]. The ability of estrogen to stimulate proteins involved in hypoxia signaling as well as to induce proangiogenic proteins may elucidate a novel role of estrogen in breast cancer neovasculogenesis. This novel physiological effect of estrogen in carcinogenesis progression is an understudied area and can shed light on the systemic activity of hormone induced cancers.

Neovasculogenesis, or the formation of new blood vessels, is modulated by estrogen and is necessary for tumor growth and sustainment. Studies using ER knockout mice observed reduced vascular repair and angiogenesis thus demonstrating the role of estrogen in vessel formation [27]. In *ex vivo* breast tissue cultures, as well as *in vivo* mouse models, E_2_ led to an increase in secretion of the proangiogenic cytokine IL-8, which is strongly correlated with the metastatic potential of breast cancer cells [28]. Further, E_2_ increased angiogenin secretion, which led to an increase in endothelial cell proliferation and was abrogated by the antiestrogen Tamoxifen [29]. In breast tumor mouse studies, E_2_ was observed to increase blood vessel formation and significantly increased endothelial progenitor cell migration to tumor sites [30]. Further, E_2_ also enhanced mRNA transcripts of proangiogenic angiopoietins 1 and 2, as well as metastatic modulating matrix metalloproteinase 2 and 9. *In vitro* models from our laboratory demonstrated E_2_ induced TG1-1 cell proliferation and migration, which was abrogated by anti-estrogens. *In vitro* tubulogenesis models have also demonstrated the role in E_2_ induced neovasculogenesis in breast cancer [30]. Considering that both hypoxia and estrogen are significant determinants of breast cancer progression and can modulate vasculogenesis processes and hence the tumor microenvironment, it is important to understand their cellular modulation so that novel intervention strategies can be examined.

This study was designed to investigate the role of estrogen on HIF-1 dependent breast cancer induced neovasculogenesis. Two types of cell lines were used: the TG1-1 murine breast cancer cell line that expresses both ERα and ERβ and the human endothelial cell line human umbilical vein endothelial cell (HUVEC). Our results define the molecular interdependence of estrogen mediated intracellular activity with hypoxia and reconnect the modulatory interdependence of cellular phenotypic changes. These studies open up new avenues of estrogen based therapeutic and preventive interventions for breast cancer that is based on the tumor microenvironment.

## Results

### Hypoxia induces HIF-1α nuclear translocation in TG1-1 cells

**Figure 1:**
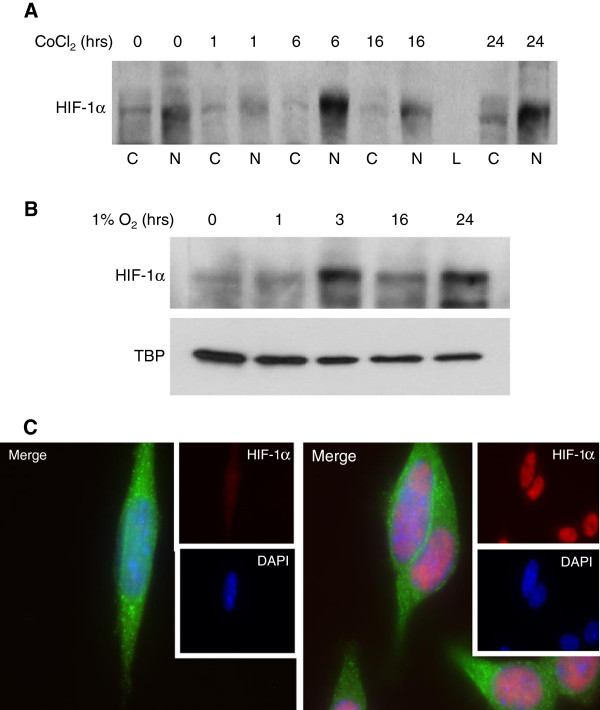
**Hypoxia induced HIF-1α nuclear translocation in TG1-1 cells is cyclical.** Western blots of TG1-1 nuclear (N) and cytoplasmic (C) lysates show induction and nuclear translocation of HIF-1α when cultured with 100μM CoCl_2_ (**A**) or in nuclear lysates of cells cultured in 1% O_2_ (**B**) with TBP as a nuclear loading control and L as a ladder lane. TG1-1 cells were also left untreated (left panel) or treated with CoCl_2_ (right panel) for 24 hours and immunofluorescently stained for VEGF (green), HIF-1α (red) and DAPI for nuclear staining (blue) and representative pictures of HIF-1α staining in TG1-1 cells also demonstrate nuclear translocation in treated cells (**C**).

First to determine whether TG1-1 cells are indeed responsive to hypoxia, we cultured cells under hypoxic conditions, specifically 1% O_2_, in a sealed hypoxic chamber for the indicated number of hours. We observed an increase in HIF-1α in nuclear lysates and used TATA binding protein (TBP) as a nuclear loading control (Figure 1A). Cells were also treated with cobalt chloride (CoCl_2_), a HIF prolyl hydroxylase antagonist, used as a positive control for HIF-1α induction (Figure 1B). HIF-1α accumulation peaked rapidly between 3-6 hours for both treatments and then returned to basal levels. To further demonstrate HIF-1α localization to the nucleus, TG1-1 cells were either untreated (left) or treated with CoCl_2_ (right) for 24 hours and stained for VEGF (green) and HIF-1α (red). The panel on the right demonstrates an increase in HIF-1α staining intensity as well as co-localization with the nuclear DAPI stain compared to the left panel with low level diffuse HIF-1α cellular staining. Together these suggest that HIF-1α is an acceptable readout of hypoxia in TG1-1 cells.

### Estrogen induces HIF-1α in breast cancer cells *in vitro*

**Figure 2:**
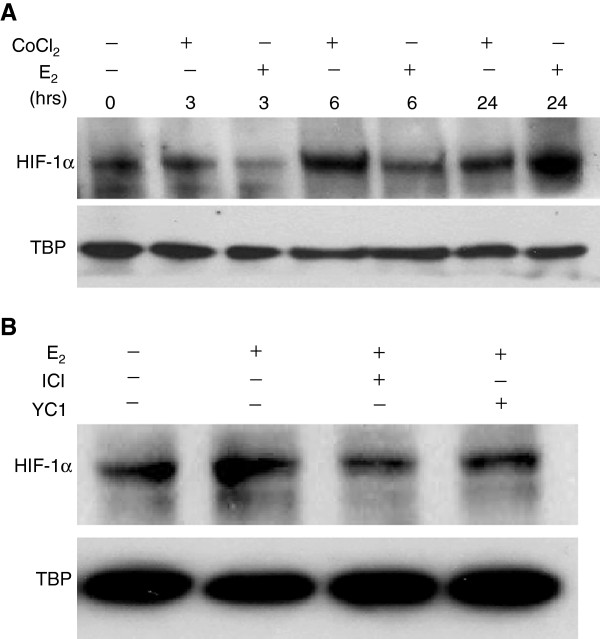
**Estrogen mediated HIF-1α translocation is sensitive to anti-estrogens and YC-1.** TG1-1 cells treated with estradiol (10^-8^ mol/L) alone show an increase in HIF-1α nuclear accumulation over time (**A**). Estradiol stimulation was validated using the ERα inhibitor Fulvestrant (ICI). TG1-1 cells were left untreated or treated for 24 hours with estradiol or estradiol plus ICI or estradiol plus the HIF-1α inhibitor YC1 (10^-5^ mol/L). HIF-1α nuclear accumulation was comparable to control levels when cells were treated with ICI or YC1 (**B**).

Recent work has focused on the oxygen independent activation of HIF-1α in hormone responsive tissues by estrogen. To verify whether estrogen was able to induce HIF-1α in breast cancer cells *in vitro,* we treated the estrogen receptor positive TG1-1 cells with E_2_ and observed an induction of HIF-1α in nuclear lysates at approximately 24 hours (Figure 2A). Further, treatment of cells for 24 hours with E_2_ and the pure anti-estrogen Fulvestrant abrogated E_2_ induced accumulation of HIF-1α comparable to cells treated with the HIF-1α inhibitor YC1 (Figure 2B) validating the E_2_ stimulation of HIF-1α.

### Estrogen induces VEGF similar to hypoxia in TG1-1 cells in a HIF-1α dependent manner

**Figure 3:**
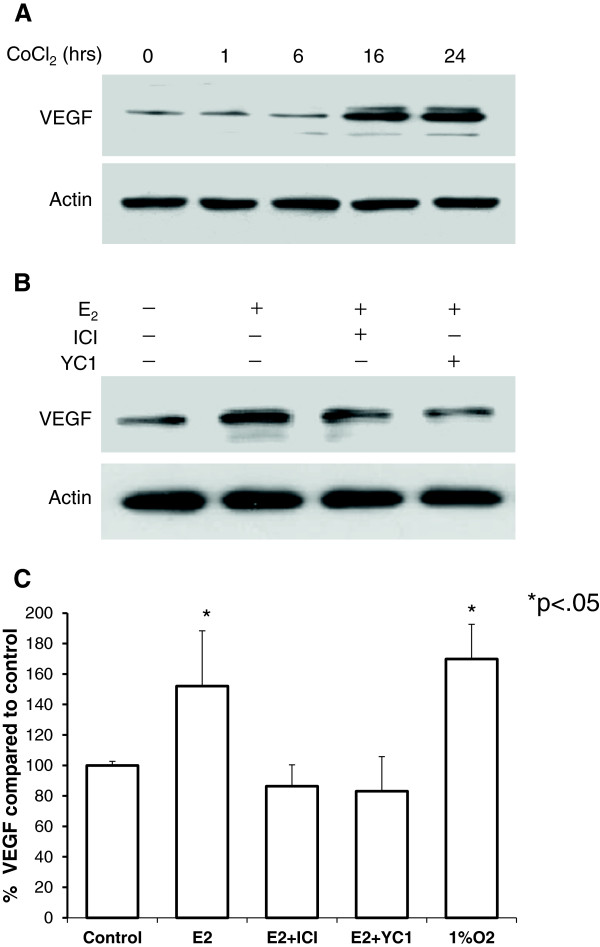
**Hypoxia and estrogen induce VEGF in TG1-1 cells.** Western blots of TG1-1 cytoplasmic lysates show that hypoxia (**A**) and estradiol (**B**) lead to an increase in VEGF. However, treatment of cells with Fulvestrant lead to a reduction in VEGF and treatment with YC-1 restored VEGF to control levels. Conditioned media from TG1-1 cells grown in 6 well plates was harvested and VEGF concentration was determined by ELISA. Corresponding cell pellets were harvested, washed, and analyzed for total protein content. Values shown are expressed as percent differences compared to control TG1-1 conditioned media normalized for the amount of protein in the cell pellet. Similarly to observed findings in the western blot analysis, cells treated with estrogen secreted significantly more VEGF when compared to control, an effect abrogated by YC-1 (**C**). Similarly cells grown under hypoxic conditions (1% O_2_) also secreted significantly more VEGF than controls. Data represents two separate experiments performed in duplicate. (*p<.05).

Stimulation of HIF-1α leads to dimerization with HIF-1β and nuclear translocation, where the heterodimer acts as a transcription factor leading to production of pro-angiogenic proteins. To test whether HIF-1α induction was functional, cytoplasmic cell lysates were isolated from TG1-1 cells treated with CoCl_2_ and E_2_ and western blots performed and probed for vascular endothelial growth factor (VEGF). Consistent with previous literature, hypoxia signaling leads to the expression of the pro-angiogenic protein VEGF in breast cancer cells *in vitro* (Figure 3A). We also observed an increase in VEGF in cells treated with E_2_ for 24 hours, an effect abrogated by Fulvestrant and more profoundly byYC1 (Figure 3B). To measure functional secretion of VEGF, we performed and ELISA and observed a marked increase in VEGF secretion when cells were treated with E_2_ or grown under hypoxic conditions (1% O_2_). Similar to western blot observation, treatment of cells with YC-1 abrogated VEGF secretion, thus demonstrating the importance on HIF-1 in estrogen induced VEGF secretion (Figure 3C). Thus, the pro-angiogenic effect of E_2_ on breast cancer cells is not solely dependent on the nuclear translocation of estrogen receptor (ER) but rather on HIF-1α translocation as well.

### Estrogen signals via the PI3K pathway leading to induction of VEGF in a HIF-1α dependent manner

**Figure 4:**
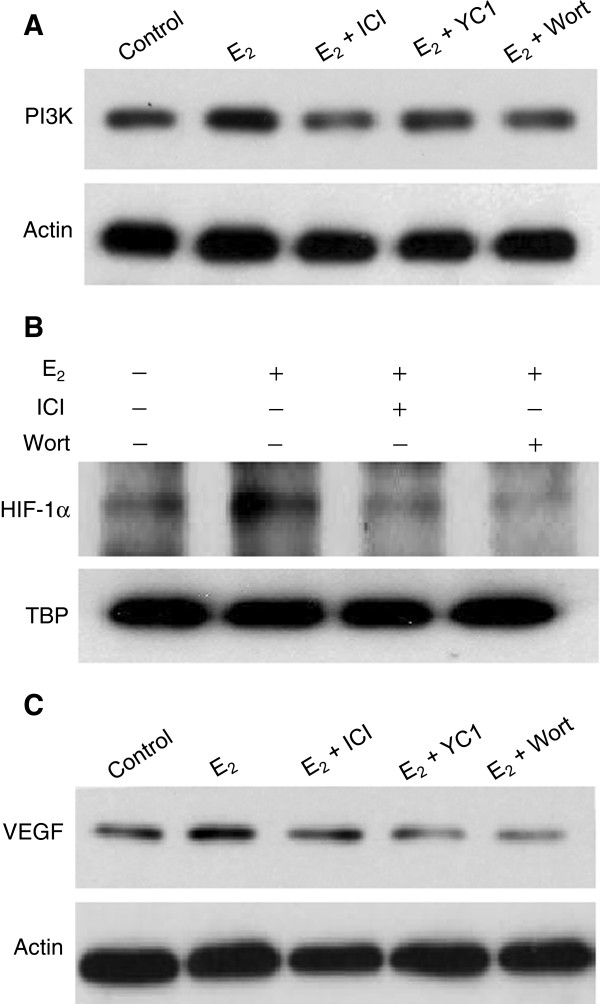
**Estrogen stimulation of HIF-1α and VEGF is PI3K dependent.** Western blots of TG1-1 whole cell lysates grown under starvation conditions show an estrogen dependent increase in PI3K (**A**) which is abrogated by Fulvestrant (ICI). The PI3K inhibitor Wortmannin prevented estradiol stimulated up regulation of HIF-1α (**B**) and VEGF (**C**).

We present evidence that E_2_ stimulation of HIF-1α and VEGF is PI3K dependent. TG1-1 cells treated with E_2_ for 24 hours show an increase in PI3K levels, an effect abrogated by Fulvestrant, further indicating functional ER signaling (Figure 4A). Treatment of TG1-1 cells with E_2_ for 24 hours in conjunction with the PI3K inhibitor Wortmannin prevented E_2_ up regulation of HIF-1α (Figure 4B). We observed the inhibition of PI3K also diminished E_2_ stimulation of VEGF in cells treated for 24 hours (Figure 4C). Thus, E_2_ signals via the PI3K pathway to stimulate HIF-1α, and inhibition of this prosurvival pathway abrogates E_2_ induction of angiogenic proteins.

### Secretion of estrogen responsive proteins via HIF-1α up regulation by breast cancer cells leads to an increase in endothelial cell migration and tubulogenesis *in vitro*

**Figure 5:**
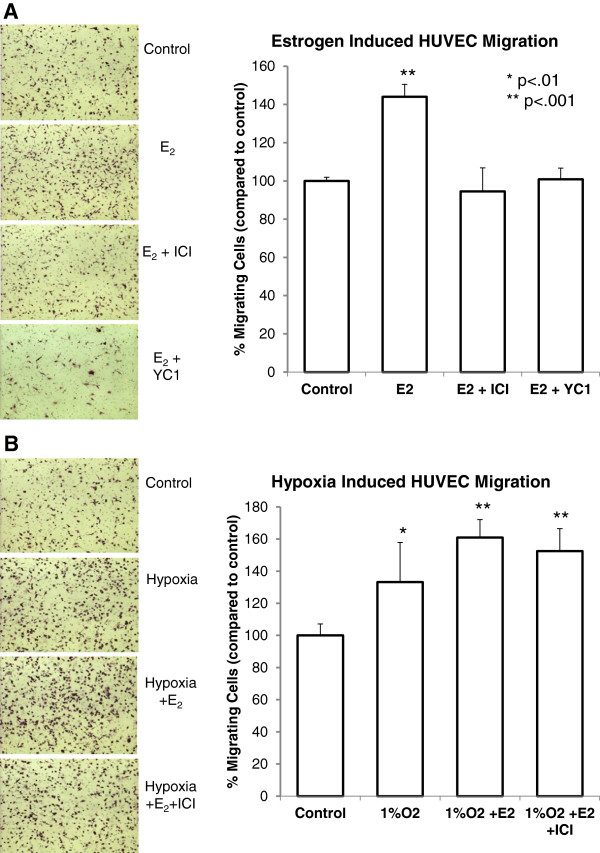
**Hypoxia and estrogen increase human umbilical vein endothelial cell (HUVEC) migration in a HIF-1α dependent manner.** Culture medium from TG1-1 cells grown under hypoxic conditions was observed to increase HUVEC migration (**A**). Further, culture media from cells treated with estradiol significantly enhanced migration (**A**,**B**) when compared to non-estradiol supplemented TG1-1 culture media, which was abrogated by YC1. * P < .001 when compared to control.

**Figure 6:**
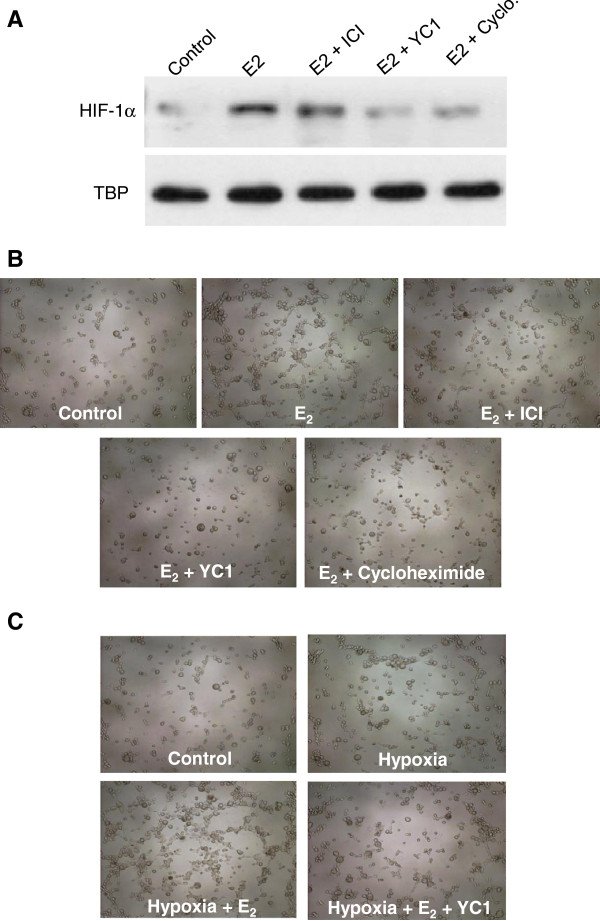
**Estrogen induced *in vitro *vasculogenesis is sensitive to anti-hypoxic and antiestrogenic agents.** TG1-1 cells were treated with estrogen in combination with the antiestrogen Fulvestrant, the anti-hypoxic agent YC-1, or 50μg/mL of the generic protein synthesis inhibitor cycloheximide. Western blot analysis of nuclear lysates demonstrated inhibition of estrogen induced HIF-1α translocation by cycloheximide (**A**). Cells were again treated as stated for 24 hours with the combination of estrogen and inhibitors prior to collection of conditioned media. Conditioned media taken from cells was used in an *in vitro* tubulogenesis assay in which secreted factors from TG1-1 cells treated with estrogen lead to HUVEC tubulogenesis. This phenotypic change was abrogated by Fulvestrant, and more strikingly by YC-1 and cycloheximide (**B**). Conditioned media from breast cancer cells grown under hypoxic conditions for 24 hours demonstrated similar effects (**C**).

Lastly, we sought to determine the cellular mechanism of estrogen induced neovasculogenesis in breast cancer progression. The process of neovasculogenesis is indispensible for tumor proliferation and metastasis and occurs largely in hypoxic tissues in which rapid tumor development quickly outgrows existing vasculature. Previously data from our laboratory demonstrated that E_2_ enhanced breast tumor neovasculogenesis *in vivo*, however the mechanism remains unclear. To address this question, culture media from TG1-1 cells treated with E_2_ with and without the anti-estrogen Fulvestrant or the HIF-1α inhibitor YC1 was used in an *in vitro* migration assay of human umbilical vein endothelial cells (HUVECs), culture media from untreated TG1-1 cells served as a control. We observed a significant increase in HUVEC migration toward the media of E_2_ treated cells, which was abrogated by Fulvestrant as well as YC1 (Figure 5A). Thus, E_2_ stimulation of HIF-1α and consequent up regulation of VEGF leads to endothelial cell migration toward breast tumor cell secreted proteins. HUVEC migration experiments in which TG1-1 cells cultured under hypoxic conditions (1% O^2^) with and without E_2_ and Fulvestrant also demonstrated only a modest synergistic effect of hypoxia and estrogen on endothelial cell migration which was not abrogated by the anti-estrogen (Figure 5B). To further characterize the role of estrogen we focused on another phenotypic characteristic of vasculogenesis *in vitro*, namely the formation of tube shaped structures by endothelial cells utilizing the tubulogenesis assay. Briefly, HUVEC cells were plated over a bed of matrigel to simulate extracellular matrix and were exposed to either control basal media or conditioned media harvested from TG1-1 cultured cells as previously mentioned. We observed an increase in tube number and length when endothelial cells were treated with tumor cell condition media from E_2_ stimulated cells (Figure 6B). Estrogen induced tubulogenesis was abrogated in the presence of the anti-estrogen Fulvestrant (ICI), the HIF-1 inhibitor YC1, and cycloheximide. Analysis of lysates from tumor cells revealed that cycloheximide treatment prevents estrogen upregulation of HIF-1α, highlighting the importance of *de novo* protein synthesis in estrogen induced vasculogenesis *in vitro* (Figure 6A). Media from TG1-1 cells grown under hypoxic conditions and concurrently treated with estrogen and inhibitors was also investigated for the impact on endothelial cell tube formation. When comparing conditioned media from tumor cells simultaneously grown under hypoxic conditions (1% O_2_) with those also treated with E_2_, we observed an increase in tubule formation which was abrogated by the HIF-1 inhibitor (Figure 6C). This further highlights the importance of both hypoxia and estrogen as determinants of tumor induced neovasculogenesis.

## Discussion

During tissue and tumor hypoxia, existing vasculature is exhausted and resident cells secrete factors including vascular endothelial growth factor (VEGF) and stromal derived factor 1 (SDF-1) with the migration of bone marrow derived endothelial progenitor cells as a significant event [31]. Endothelial cell migration, however, is reliant on expression of cell surface receptors such as VEGFR1 and 2, and CXCR-4, which bind VEGF and SDF-1 respectively. During rapid tumor development tumor and stromal cells create an angiogenic milieu conducive to rapid expansion of the vasculature. We had earlier demonstrated that vasculogenesis is an estrogen mediated phenomenon [30]. A decrease in oxygen tension is a significant determinant of tumor progression and tumors rapidly adapt to their changed metabolic intracellular milieu however as evidenced by this study systemically they continue to mimic physiological processes such as estrogen mediated activity. The molecular mechanisms by which estrogen and hypoxia (low oxygen tension) impact tumor development are only recently being uncovered.

Numerous studies have implicated hormones in the activation of hypoxia inducible factor 1 (HIF-1). HIF-1 is a heterodimeric transcription factor composed of the oxygen sensitive alpha subunit and the constitutively present beta subunit. We have demonstrated a functional HIF-1 protein in our mouse mammary tumor cell line TG1-1. TG1-1 cells are responsive to both the known HIF-1 stabilizer cobalt chloride as well as hypoxic culture conditions, specifically 1% O_2_, in which we observed stabilization of the HIF-1α subunit and its subsequent translocation into the nucleus. However a decrease in oxygen concentration is not the only activator of HIF-1 stabilization and translocation as demonstrated by us in this study and elsewhere. He *et al.* found that in obesity models, insulin was able to up regulate both HIF-1 mRNA and protein levels in a PI3K/mTOR dependent manner [32]. The interdependence of estrogen mediated cellular activity and hypoxia have been observed in various other cellular models. Kazi and Koos showed that in a rat uterine model, estrogen treatment lead to up regulation of VEGF, and found that both ERα and HIF-1α were recruited to the VEGF promoter. Further, they identified that the PI3K pathway was essential for this phenomenon [22]. Hua *et al.* further demonstrated that E_2_ treatment leads to up regulation of HIF-1α in the ovarian cancer cell lines ES-2 and SKOV3 in a time dependent manner, peaking around 24 hours [23]. In agreement with this finding, we also observed in increase in HIF-1α levels in our breast cancer cells at 24 hours treatment. This was interesting as the hypoxia mimetic, cobalt chloride, induced a much more rapid HIF-1α response. This finding may highlight an indirect role of estrogen in HIF-1α up regulation in which estrogen regulated proteins may lead to HIF-1α increases in an autocrine fashion. This secretory autocrine loop was demonstrated in androgen induced prostate cancer cell lines and thus may be functionally equivalent in many different hormone responsive tissues [33]. These studies establish a link between estrogen mediated signal transduction and hypoxia in co-operating to regulate angiogenic factors. Earlier we had established that breast cancer induced tumorigenesis required the presence of endothelial progenitor cells and that the process of vasculogenesis was both tumor induced factor modulated and at the systemic level regulated by estrogens. Since hypoxia is the most common metabolic adaptation of rapidly proliferating breast cancer we attempted in this study to define the molecular link of hypoxia and estrogen.

Our studies clearly indicate that hypoxia mimics estrogen mediated function as determined by estrogen’s ability to regulate HIF-1α and VEGF, both of which are molecular mediators of hypoxic condition. The release of angiogenic factor VEGF had functional significance as evidenced by the ability of E_2_ treated TG1-1 conditioned media to enhance both migration and tubulogenesis which was responsive to antiestrogens and anti-hypoxia agents. We also provide evidence that inhibition of the E_2_-dependent PI3K up regulation inhibited HIF-1α translocation. This molecular interdependence was translatable at the cellular phenotypic level as both migration and tubulogenesis of endothelial cells were responsive to antiestrogens and anti-hypoxia agents.

Paracrine/autocrine protein secretion is important for both tumor cell and endothelial cell migration during tumor progression and metastasis. Voss *et al.* highlighted the importance of hypoxia mediated protein secretion in migration of breast cancer cells lines MCF-7, MDA-MB-231, MDA-MB-435S, and MDA-MB-468 using conditioned media and found that media of hypoxic cells increased migration of normoxic cells. Also, media harvested from hypoxic cells lead to an increase in neutrophil granulocyte migration [34]. Similarly, Fujiwara *et al.* found hypoxia increased migration and invasiveness of glioma cell lines via up regulation of MMP-2 and a corresponding down regulation of TIMP-2 [35]. Further, HIF-1 induction also leads to expansion of glioma stem cells, which is dependent on Akt/ERK signaling [36]. Perhaps the most documented role of hypoxia pertains to its importance in directing endothelial cell migration. Meininger, Shelling, and Granger noted that bovine aortic and coronary endothelial cells proliferated when exposed to 2% O_2_ and that this proliferation was most likely due to hypoxia mediated adenosine secretion [37]. As early as 1992, Shweiki *et al.* observed a hypoxia dependent increase in VEGF in glioblastoma multiforme *In situ* in which cells spatially closer to necrotic centers produced more VEGF and that correspondingly more capillaries clustered near these VEGF producing cells [38]. Other studies have observed the same phenomenon in other tumors including breast in which an increase in VEGF mRNA levels and small blood vessels were located in close proximity to ductal carcinoma *in situ*, infiltrating ductal carcinoma, and metastatic ductal carcinoma tumors when compared to normal or non-malignant breast tissue [39]. In this and our previously published study we provide experimental evidence that estrogen and tumor derived angiogenic factors not only recruit endothelial progenitor cells and induce neovasculogenesis but that also established (as evidenced by the use of HUVEC cells) endothelial cell migration and tubulogenesis can be modulated by estrogen and hypoxic conditions. The observation that inhibition of the signal transduction pathway of estrogen can affect hypoxia and that anti-hypoxic agents can be interchangeably used with antiestrogenic agents in modulating both angiogenic processes and tumor phenotype opens up a novel intervention avenue that is molecular target based modulation of tumor microenvironment that is directed toward cell-cell interactions.

## Conclusions

The complexity of the tumor microenvironment and the redundancy of the signaling pathways involved cannot be underestimated. Our data provides evidence that estrogen can molecularly mimic hypoxia by activating HIF-1α and that estrogen mediated HIF-1α induction requires a functional PI3K signaling pathway. This active interdependence presumably co-operates to produce an angiogenic environment conducive to endothelial cell migration and vasculogenesis. The continued hypoxic conditions in the tumor may lead to eventual ‘estrogen-independent’ cell type that at the signal transduction level produced estrogen inducible elements constitutively. These studies clearly suggest the need to test a combination of anti-estrogenic and anti-hypoxic agents as an intervention strategy for breast cancer prevention and therapy.

## Materials and methods

### Cell culture

The carcinoma cell line used for this study was TG1-1, a mouse mammary epithelial cell line, and the primary human endothelial cells HUVECs (human umbilical vein endothelial cells). TG1-1 was cultures in DMEM (Mediatech, Herndon, VA) supplemented with 10% fetal bovine serum (FBS) (Atlanta Biologicals, Atlanta, GA), penicillin 10,000 IU/mL, streptomycin 10,000 μg/mL (Mediatech) and 2mM L-glutamine (Mediatech). HUVEC (human umbilical vein endothelial cell) cells were obtained from American Type Culture Collection (ATCC) (Manassas, VA) and grown in F12K (Mediatech) supplemented with 10% FBS, 0.1mg/mL Heparin, 0.03mg/mL endothelial cell growth supplement (Sigma Aldrich). Cells were grown at 37°C in a humidified atmosphere with 5% CO_2_ unless otherwise noted. For the cellular factors studied in this manuscript, the estrogen dependent, hypoxia induced change TG1-1 cells respond to, specifically producing VEGF, were similar to human breast cancer cells and hence the interdependent interaction of TG1-1 and HUVEC was rationalized.

For experiments, TG1-1 cells were grown to 75-80% confluence and serum-starved overnight in phenol red-free DMEM (Mediatech) supplemented with penicillin and streptomycin. For hypoxia experiments cells were grown in 1% O_2_ in a modular incubator chamber (Billups-Rothenberg Inc.). Addition of E_2_ to breast cancer cells was always on serum starved cells so as to define the estrogenic mediated increase in protein expression.

### Western blot analysis

Cells were harvested using 0.25% trypsin (Mediatech), washed with PBS, and lysed (1X10^6^/100μL of lysis buffer) using the radioimmunoprecipitation assay (RIPA) buffer [50mM Tris–HCl (pH 7.4), 150 mM NaCl, 0.2% sodium deoxycholate, 0.1% SDS, 0.5% NP40, 1mM Pefabloc] and incubated on ice for 30 minutes with vortexing every 5 minutes. Samples were centrifuged at 14,000 rpm for 30 minutes at 4°C then supernatants collected for whole cell lysates. For nuclear/cytoplasmic isolation we used the NE-PER Nuclear and Cytoplasmic Extraction Kit from Thermo Scientific and followed manufacturer’s directions. Cell lysates (10-20μg) were subjected to 10% SDS-PAGE under reducing conditions (presence of β-mercaptoethanol) as previously described. Proteins were transferred to Immobilon-P membranes at 220 mA for 2 h and membranes were blocked in 5% dried milk in TBST [200mM Tris–HCl, pH 7.4, 150mM NaCl, and 0.01% Tween-20 added fresh/liter of 1X TBS (TBST)] for 2 h at room temperature on a shaker. After blocking, membranes were incubated overnight at 4°C with either HIF-1α (Abcam, Cambridge, MA), TBP (Abcam), VEGF (Santa Cruz Biotechnology, Santa Cruz, CA), Actin (Santa Cruz), or PI3K (Cell Signaling Technology) antibody in TBST. Membranes were subsequently washed three times in TBST and incubated with the respective horseradish peroxidase (HRP) conjugated secondary antibody (Pierce, Rockford, IL), for 2 h at room temperature in TBST containing 2% dried milk. Membranes were then washed three times with TBST and developed using ECL substrate (Pierce) and detected on Denville autoradiography film.

### VEGF Enzyme-linked Immunosorbent Assay (ELISA)

The RayBio Mouse VEGF Quantikine ELISA Kit (RayBiotech, Inc.,) was used to quantitate the levels of VEGF in conditioned media obtained from TG1-1 cells according to manufacturer’s instructions. ELISA was performed on each sample in duplicate. Protein content of cell pellets was determined in duplicate by using the Bradford protein assay (Bio-Rad).

### Transwell migration assay

BD Biocoat Control inserts (BD Biosciences, Bedford, MA) with 8-μm pore membrane filters were used for migration experiments as previously described. Briefly, TG1-1 cells were serum starved overnight for 16 h; the media was then replaced with serum free, phenol red-free DMEM (Mediatech) supplemented with ± 10^-8^ M Estradiol (Sigma Aldrich), ± 10^-6^ M Fulvestrant (Sigma), ± 10^-5^ M YC-1 and culture media was subsequently harvested 24 h later. Conversely, TG1-1 cells were incubated under hypoxic conditions and media collected after 24 h. HUVEC cells were then harvested by trypsinization and 2.5 x 10^4^ cells were seeded in the upper chamber in 500 μl of serum-free, phenol red-free DMEM. The lower chamber contained 750 μl of the harvested TG1-1 media. After 18 h of incubation non-adherent cells were removed from the upper chamber using a cotton swab. Migrant cells on the lower surface were then fixed with 100% methanol and stained using 1% toluidine blue, 1% borax stain and then washed twice in distilled water. Inserts were allowed to dry and then visualized under 10x magnification. Experiments were performed in duplicate and data represents number of migrating cells per 10x field and normalized to cell counts of control treatment groups.

### Endothelial tube formation assay

96 well plates were coated with 100 μl of growth factor reduced, phenol red-free Matrigel (BD Biosciences). HUVEC cells were harvested by trypsinization then added at a concentration of 10,000 cells/well in serum-free, phenol red-free DMEM or TG1-1 cell conditioned culture media as previously described. Plates were then incubated at 37°C for 4-6 h and visualized using 5x magnification, images were obtained using Axiovert 4.0.

### Immunoflourescence staining

TG1-1 cells were harvested as described and seeded at a density of 1 x 10^4^ into 8 well chamber slides (Becton Dickson) in complete DMEM and were allowed to adhere 24 h. Media was then removed and replaced with serum-free, phenol red-free DMEM and cells were serum starved overnight. Starvation media was removed and replaced with DMEM supplemented with ± 10^-8^ M E2 ± 10^-6^ Fulvestrant ± 10^-5^ YC1 or grown under hypoxic conditions. Media was then removed and cells washed three times with phosphate buffered saline (PBS). Cells were then fixed with 4% para-formaldehyde at room temperature for 15 minutes then washed again three times with PBS. Cells were then permeabilized with 0.5% Triton-X for 5 min at room temperature and again washed three times with PBS. Cells were then blocked in 0.2% Triton-X, 10% goat serum (Sigma) and 3% bovine serum albumin (BSA) for 30 min at room temperature. Cells were then incubated overnight at 4°C with either HIF-1α or VEGF antibody in 1% BSA. Wells were then washed three times with PBS and incubated with the respective secondary antibody conjugated to either Alexa-fluor 488 or 595. Images were gathered using the Axiovision Rel 4.8 program under 40x magnification on the Axiovert 200M microscope (Carl Zeiss MicroImaging Inc., Thornwood, NY).

### Statistical analysis

The data presented here represent three replicates. Statistical analysis was performed using the Student’s *t*-test. Differences were considered statistically significant at P<0.05. *=P<.001.

## Abbreviations

BM-EPC: Bone marrow derived endothelial progenitor cell; E_2_: 17-β-estradiol; VEGF: Vascular endothelial growth factor; HIF-1: Hypoxia inducible factor 1; YC-1: (3-(5’-hydroxymethyl-2’-furyl)-1-benzylindazole); HUVEC: Human umbilical vein endothelial cell; PI3K: Phosphatidylinositol 3 kinase; ERα/β: Estrogen receptor alpha/beta; ERE: Estrogen responsive element; TNF-α: Tumor necrosis factor alpha; ARNT: Aryl hydrocarbon receptor nuclear translocator; SDF-1: Stromal derived factor 1; MAPK: Mitogen-activated protein kinase; IL-8: Interleukin 8; CoCl_2_: Cobalt chloride; TBP: Tata binding protein; VEGFR1/2: Vascular endothelial growth factor receptor 1/2.

## Competing interests

The authors declare that they have no competing interests.

## Authors' contributions

ALG contributed to conception and design of the study, carried out western blot, immunoflourescent, migration, and tubulogenesis studies, and contributed to drafting of the manuscript. SR participated in acquisition of data and drafting of the manuscript, RS participated in analysis and interpretation of the data and revising of the manuscript/contributing to intellectual content, AM provided reagents/analysis tools and contributed to intellectual content of the manuscript, RKT helped conceive the study, participated in design and coordination of the study and drafting the manuscript. All authors read and approved the final manuscript.
